# Alien Chromosome Serves as a Novel Platform for Multiple Gene Expression in *Kluyveromyces marxianus*

**DOI:** 10.3390/microorganisms13030509

**Published:** 2025-02-25

**Authors:** Yilin Lyu, Jungang Zhou, Yao Yu, Hong Lu

**Affiliations:** 1 State Key Laboratory of Genetic Engineering, School of Life Sciences, Fudan University, Shanghai 200438, China; 18210700033@fudan.edu.cn (Y.L.); zhoujg@fudan.edu.cn (J.Z.); 2 Shanghai Engineering Research Center of Industrial Microorganisms, Shanghai 200438, China

**Keywords:** alien chromosome, *Kluyveromyces marxianus*, *Saccharomyces cerevisiae*, *cis*-regulatory elements, expression of heterologous proteins

## Abstract

*Kluyveromyces marxianus* is an emerging yeast cell host for diverse products, but multiple-gene expression in *K. marxianus* faces challenges due to limited current knowledge of *cis*-regulatory elements and insertion loci. Our previous study transferred an alien *Saccharomyces cerevisiae* chromosome I (R1) into *K. marxianus*, resulting in the creation of the monochromosomal hybrid yeast KS-R1. All R1 genes were actively transcribed, providing a series of loci with varying transcriptional activities. Here, we explore the use of R1 as a novel platform for stable, multi-gene integration and expression. By deleting three essential *K. marxianus* genes while complementing their functions with orthologs on R1, we achieved stable propagation of R1 in the absence of selective pressure. We characterized several loci on R1 that exhibit stable transcriptional activities under various conditions. *GFP* inserted in place of genes at six such loci demonstrated varying expression levels. Strains with *GFP* at two loci exhibited significantly higher expression than those with *GFP* at a single locus. Furthermore, we replaced five R1 genes with disulfide bond formation genes from *Pichia pastoris* at distinct loci, resulting in the active expression of all five genes and significantly enhanced production of heterologous glucoamylases BadGLA and TeGlaA. Our findings demonstrate that alien chromosomes offer a stable and versatile platform for the coordinated expression of multiple heterologous genes, serving as valuable tools for metabolic engineering and synthetic biology.

## 1. Introduction

*Kluyveromyces marxianus* (Km) is a homothallic budding yeast belonging to the *Saccharomyces* subclade within hemiascomycetes. Km harbors various favorable features for industrial production, including “generally regarded as safe” (GRAS) status, thermotolerance, fast growth, and a broad spectrum of utilizable carbon sources [1,2,3]. Since 1980, Km has become an emerging yeast cell host for diverse products. To date, over 50 recombinant proteins, including industrial enzymes, virus-like particles, and food additive proteins, have been successfully expressed by Km [4,5,6,7], with the highest yield reaching 16.8 g/L [8]. The highest titers of bioethanol produced by Km reached 110 g/L [9]. Km has also achieved the production of various chemicals, such as lactic acid [10], xylitol [11], 2-phenylethanol [12], hexanoic acid [13], astaxanthin [14], and polyketides [15]. Apart from that, Km possesses a high potential to produce aroma compounds, including fruit esters, furanones, ketones, carboxylic acids, and aromatic hydrocarbons [16,17].

Genetic engineering of Km always involves the regulated expression of multiple heterologous genes. To display the cellulosome on the cell surface, five fungal cellulase genes were inserted into the genome of Km [18]. To increase the yield of soy leghemoglobin, six heterologous heme synthesis-related genes were introduced into Km via yeast artificial chromosomes (YAC) [7]. To remodel the disulfide bond formation pathway, seven *Pichia pastoris* genes were introduced into Km using YAC [8].

The current bottleneck in multi-gene expression in Km lies in the lack of knowledge about the *cis*-elements with varying transcriptional activities, which have not been as well-characterized as they have been in Sc, particularly promoters and terminators. Previous studies have identified a series of native Km promoters with varying transcriptional activities, such as *TEF3* (strong), *PDC1* (strong), *GPD1* (medium), *INU1* (medium), *MED4* (weak), and *SRB7* (weak) [19,20,21]. However, native promoters, when placed upstream of heterologous genes, might cause recombination with native loci, leading to genetic instability. Additionally, native promoters may compete for transcriptional factors, affecting the functions of native genes. Heterologous promoters can reduce the risk of recombination and competition for trans-acting factors. However, only a few heterologous promoters, including the *Saccharomyces cerevisiae* (Sc) *ADH1*, *ADH2*, *TEF1*, *PGK1*, *TDH3*, and *GAL1* promoters; the *Pichia pastoris AFT1* promoter; and the *Ashbya gossypii TEF* promoter, have been identified as functional in Km [15,19,20,21,22]. Moreover, all these heterologous promoters are strong or medium promoters, which cannot meet the demand for the expression of multiple genes at various levels. Terminators provide additional means to fine-tune gene expression [20]. A series of Km terminators have been characterized, but these terminators might cause undesirable recombination with native loci [20]. A few heterologous terminators, including the *A. gossypii TEF* terminator, the *ScADH1* terminator, and some short synthetic terminators, have been proven to be functional in Km [21,23]. The limited number of available heterologous terminators hinders multiple-gene engineering in Km.

The limited availability of insertion loci is another problem for multiple heterologous gene expression. The insertion of heterologous genes at genomic loci exhibits positional effects, which might interfere with the expression of the inserted genes, as well as the expression of contextual genes around the locus [24]. Plasmids provide episomal loci for gene insertion, but the stability of plasmid propagation is low [25,26]. Yeast artificial chromosomes of Km (KmYACs) exhibit high stability comparable to natural chromosomes and can carry multiple genes without affecting the native genome [8]. However, the YAC backbone lacks *cis*-elements for integrated genes. Gene cassettes, including promoters, open reading frames (ORFs), and terminators, have to be assembled before being integrated into the YAC [8]. Therefore, the issue of insufficient *cis*-elements still needs to be addressed during the insertion of multiple genes into YACs.

Our previous study reported the transfer of alien Sc chromosomes 1 (R1) or 3 (R3) into Km, resulting in the creation of the monochromosomal hybrid yeasts KS-R1 and KS-R3, respectively [27]. R1 and R3 can replicate and segregate normally in KS-R1 and KS-R3, respectively. The introduction of R1 and R3 did not cause any noticeable growth defects. R1 and R3 contain 89 and 151 genes, respectively, all of which can be expressed in the hybrids. Since the Sc chromosomes are heterologous, the genes on them are dispensable for the hybrid and can be safely disrupted or deleted. Therefore, the gene loci on the alien chromosomes can serve as safe integration sites for heterologous genes. Moreover, the expression levels of R1 and R3 genes were diverse in the hybrid, thus providing groups of loci with different transcriptional activities.

Here, by using KS-R1 as a model, we investigate the strategy of utilizing an alien chromosome from *S. cerevisiae* as a platform for multiple-gene insertion and expression. Three essential Km genes were deleted while the growth of the hybrid was maintained by their orthologous genes on R1. The resulting strain exhibited stable propagation of R1 without selective pressure and provided markers for subsequent engineering. We characterized loci on R1 that exhibit stable expression levels under different conditions. *GFP* was inserted into six stable loci to determine the varying expression levels among loci. Strains harboring *GFP* at two loci showed markedly higher expression compared to those with *GFP* at a single locus. Finally, five disulfide bond formation genes from *P. pastoris* were inserted into five different loci on R1. All five genes were actively expressed and significantly enhanced the expression of heterologous proteins.

## 2. Materials and Methods

### 2.1. Strains, Media and Plasmids

The yeast strains used in this study are listed in Supplementary Table S1. The plasmids used in this study are listed in Supplementary Table S2, and primers are listed in Supplementary Table S3. The following media were used in this study: YPD (2% peptone, 1% yeast extract, 2% glucose, 2% agar for plates), YD (2% yeast extract, 4% glucose), supplemented synthetic medium lacking uracil (SC-Ura) (0.67% yeast nitrogen base without amino acids (291930, BD, Franklin Lakes, NJ, USA), 2% glucose, 2 g/L DO Supplement-Ura (630416, Takara, Beijing, China), 2% agar for plates), and SC medium (SC-Ura medium supplemented with 200 mg/L uracil, 2% agar for plates). Nourseothricin (96736-11-7, RuiTaiBio, Beijing, China) and 5-FOA (F8420, Solarbio, Beijing, China) were added to YPD medium at final concentrations of 100 mg/L and 400 mg/L, respectively, to prepare YPDN and YPD + 5-FOA media. The recipe for SC-Ura + GH plates is as follows: 0.17% yeast nitrogen base without amino acids and ammonium sulfate (233520, BD, Franklin Lakes, NJ, USA), 0.1% glutamate (L817833-500g, Macklin, Shanghai, China), 2% glucose, 2 g/L DO Supplement-Ura, 200 mg/L G418 (A600958-0005, Sangon, Shanghai, China), 300 mg/L hygromycin B (H8080, Solarbio, Beijing, China), and 2% agar.

The construction of FIM-1ΔU was described previously [25]. The engineered chromosome 1 of Sc strain W303-1A was transformed into FIM-1ΔU to obtain KS-R1 [27]. Other strains used in this study were constructed using a CRISPR/Cas9 strategy with an ARS1-based CRISPR plasmid LHZ531 [23] or LHZ1660. Primers containing a 20 bp target sequence were annealed and inserted into the SapI site of LHZ531 to obtain CRISPR plasmids LHZ1654, LHZ1655, LHZ1657, and LHZ1659. The open reading frame (ORF) of *natMX4*, along with the *TEF* promoter and terminator amplified from pFA6a-A1:natMX4 (Addgene: 134428), was inserted into the SpeI and SmaI sites of LHZ531 to obtain CRISPR plasmid LHZ1660. Primers containing a 20 bp target sequence were annealed and inserted into the SapI site of LHZ1660 to obtain CRISPR plasmids LHZ1650-LHZ1653, LHZ1656, and LHZ1658. To obtain a donor sequence for gene deletion, 500 bp upstream and downstream sequences of the gene to be deleted were amplified and ligated by overlap PCR. KS-R1 was co-transformed with LHZ1650 and the *hyg^R^*Δ donor using the lithium acetate method [28], and transformants were selected in YPDN medium. A positive clone was named KS-R1-Δh. Similarly, *kan^R^* was deleted in KS-R1-Δh to obtain KS-R1-Δhk using LHZ1651. *KmPOP5* and *KmPRP45* were deleted together in KS-R1-Δhk to obtain KS-R1-Δhkpp using LHZ1652. *KmMAK16* was deleted in KS-R1-Δhkpp to obtain KS-R1-Δhkppm using LHZ1653. *URA3* was deleted in KS-R1-Δhkppm by counterselection on YPD + 5-FOA plates to obtain KS-R1E.

To obtain the donor sequence to replace an R1 gene with *GFP*, 500 bp upstream and downstream sequences of the target gene were fused with the 5′ end and 3′ end of the *GFP* ORF by overlap PCR. To replace *CYS3* with *GFP*, KS-R1E was co-transformed with LHZ1654 and the *GFP* donor, and transformants were selected in SC-Ura medium. Similarly, *PAU7*, *FLC2*, and *ADE1* were replaced with *GFP* using LHZ1655, LHZ1657, and LHZ1659, respectively. To replace *ERV46* with *GFP*, KS-R1E was co-transformed with LHZ1656 and the *GFP* donor, and transformants were selected in YPDN medium. Similarly, *LDS1* was replaced with *GFP* using LHZ1658. To construct strains with *GFP* at two loci, in KS-R1E/*lds1*Δ::*GFP*, either *CYS3* or *ERV46* was replaced with an additional *GFP* using the CRISPR plasmid, as mentioned above. The donor sequences to replace R1 genes with *P. pastoris* genes were constructed in the same manner as *GFP* donors. In KS-R1, *FLC2* was replaced with *PpYPT1* using CRISPR plasmid LHZ1657. Subsequently, *LDS1* was replaced with *PpMPD1-2* using LHZ1658. Next, *PAU7* was replaced with *PpPDI1* using LHZ1655. Next, *CYS3* was substituted with *PpMPD1-1* via LHZ1654, and finally, *ERV46* was replaced with *PpMPD1-3* using LHZ1656. The CRISPR plasmid was removed before the next gene replacement. The substitution efficiency at each step was measured. The resultant strain containing five substitutions was named KS-R1E5P.

Plasmids for expressing BadGLA (LHZ1020) and TeGlaA (LHZ1021) were described previously [8].

### 2.2. Growth Curves and Chromosome Stabilities

KS-R1, KS-R1-Δhkpp, and KS-R1-Δhkppm were cultured in SC-ura liquid medium, while KS-R1E was cultured in YPD liquid medium overnight. The overnight cultures were then diluted into YPD medium to an initial OD_600_ of 0.2 and grown at 30 °C for 72 h, during which OD_600_ measurements were recorded. To assess the chromosome stability of KS-R1 and KS-R1E, the cultures were cultivated at either 30 °C or 37 °C. Every 24 h, the cultures of KS-R1 and KS-R1E were diluted into fresh YPD medium with an initial OD_600_ of 0.2. After five days of continuous passage (approximately 7 generations per day), the cultures of KS-R1 were diluted and plated onto YPD and SC-Ura + GH plates. The stability of R1 was determined by dividing the colony-forming unit count on SC-Ura + GH plates by that on YPD plates. For KS-R1E, the maintenance of R1 in eight different clones was verified by PCR of seven position markers.

### 2.3. RNA-Seq Data Analysis

RNA-seq data were extracted from a previous study [27]. Cells were grown in SC-Ura + GH medium overnight, then diluted into 50 mL fresh YPD medium to an initial OD_600_ of 0.2. When the OD_600_ reached 0.6, the cells were collected and washed with ddH₂O. To induce stress, the medium was supplemented with tunicamycin at a final concentration of 1 μg/mL or NaCl at a final concentration of 1 M. The cells were treated for 1 h, collected, washed with ddH_2_O, and then subjected to RNA extraction and RNA-seq [27]. To identify gene loci exhibiting stable transcriptional activities, the coefficients of variation (CV) were calculated by dividing the standard deviation by the mean. Sc-specific genes were identified previously [27]. The relative FPKM level refers to the ratio of the average FPKM value of an R1 gene across three conditions to the average FPKM value of three housekeeping genes (*SWC4*, *TRK1*, *MPE1*) across the same conditions.

### 2.4. Measurement of GFP Gene Expression

Cells were grown in SC medium overnight, then diluted into fresh SC medium to an initial OD_600_ of 0.2. To induce stress, the medium was supplemented with tunicamycin at a final concentration of 0.2 μg/mL or NaCl at a final concentration of 0.5 M. Cells were then cultured at 30 °C and 220 rpm for 24 h. A total of 100 μL of culture was collected, and the fluorescence intensities were measured at 485–528 nm using a Synergy2(BioTek, Winooski, VT, USA). The relative green fluorescence intensity was calculated as the ratio of green fluorescence intensity to OD_600_. For the subculturing of cells with *GFP* at two loci, an overnight culture was diluted into fresh SC medium with an initial OD_600_ of 0.2, and the cells were then grown at 30 °C. Dilution was performed every 24 h. After three days of continuous passage, the culture was collected to measure GFP intensity. The culture was also restreaked onto YPD medium to obtain single clones for PCR assays. The experiments were performed with three parallel cultures.

### 2.5. qPCR

KS-R1E strains with *GFP* integrated at different loci and KS-R1E5P were cultured overnight in YPD liquid medium. The cultures were diluted into YPD to an initial OD_600_ of 0.2. After 12 h of cultivation, 1 mL of the cultures was collected and washed with ddH_2_O. Total RNA was extracted using the ZR Fungal/Bacterial RNA MiniPrep kit (R2014, ZymoResearch, Irvine, CA, USA). Samples were reverse transcribed using HiScript III All-in-One RT SuperMix Perfect for qPCR (R333-01, Vazyme, Nanjing, China). The qPCR was performed using a ChamQ Universal SYBR qPCR Master Mix (Q711-02, Vazyme, Nanjing, China). The mRNA level of a specific gene was normalized to the mRNA level of a housekeeping gene, *SWC4*. The primers used in qPCR are listed in Supplementary Table S3. The experiments were performed with three parallel samples.

### 2.6. Enzymatic Assays

KS-R1E was transformed with LHZ1020 or LHZ1021, and transformants were selected on SC-Ura plates. Cells were grown in YD liquid medium for 72 h. We collected 100 μL of supernatant for enzymatic assays. The enzymatic activities in the supernatant were determined as described previously [8]. In brief, the activities of BadGLA and TeGlaA were determined by using 1% (*w*/*v*) soluble starch as a substrate. A total of 5 µL of diluted supernatant was mixed with 45 µL of 20 mM NaAc (pH 5.5) and 50 µL of 1% soluble starch in 20 mM NaAc (pH 5.5). After 5 min incubation at 60 °C, 100 µL of DNS (D7800, Solarbio, Beijing, China) was added, and the mixture was incubated at 98 °C for 5 min. Absorbance at A540 was then measured with 100 µL of the sample. One unit of activity of these enzymes was defined as the amount of enzyme required to release 1 µmol glucose per minute [21].

### 2.7. Statistical Analysis

Each assay subjected to statistical analysis comprised three biological replicates. Statistical significance was determined using a *t*-test. A *p*-value of < 0.05 was considered statistically significant.

## 3. Results

### 3.1. Maintenance of R1 by Complementation Between Essential Genes on R1 and Deletion of Their Counterparts in K. marxianus

**Figure 1:**
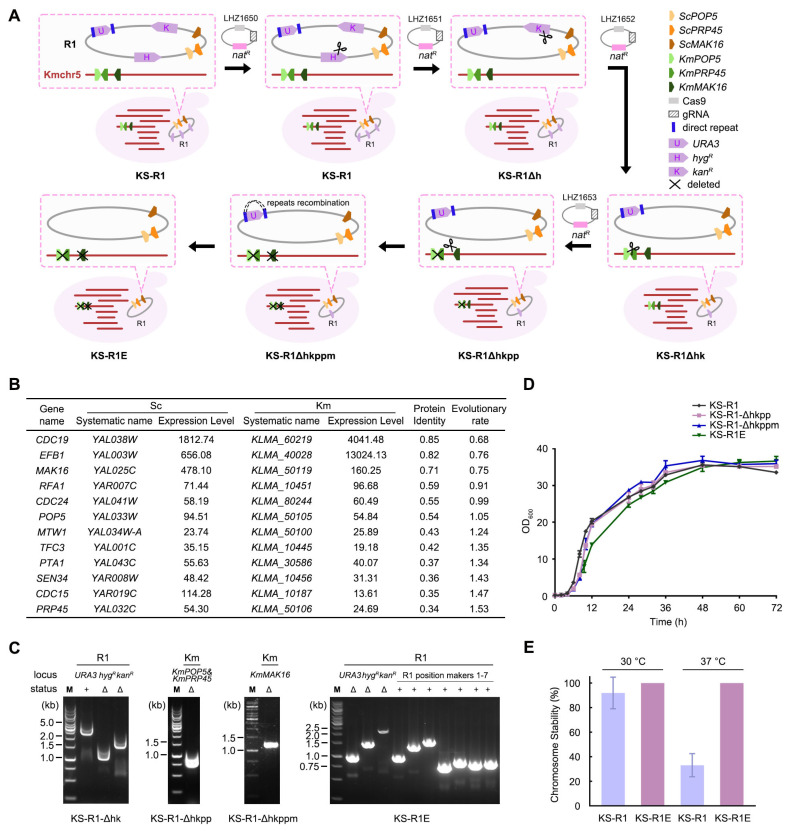
Complementation between essential genes on R1 and deletion of their counterparts in *K. marxianus*. (**A**) Schematic view of R1 engineering. (**B**) List of Sc essential genes on R1. Expression levels were indicated by read counts in the transcriptomic analysis. The evolutionary rate was obtained from OrthoDB [31]. (**C**) Identification of genotypes for KS-R1-Δhk, KS-R1-Δhkpp, KS-R1-Δhkppm, and KS-R1E. Three selective markers on R1 (*URA3*, *kan^R^*, *hyg^R^*), seven R1 position markers, as well as the *KmPOP5*, *KmPRP45*, and *KmMAK16* loci in the Km genome were characterized by PCR. The positions of primers relative to the loci and the expected sizes of the PCR products are shown in Figure S1A. (**D**) Growth curves of KS-R1, KS-R1-Δhkpp, KS-R1-Δhkppm, and KS-R1E in YPD medium. Cells were grown in YPD for 72 h, and OD_600_ was measured at the indicated times. Values represent the mean (*n* = 3). (**E**) Stability of R1 in KS-R1 and KS-R1E. Cells were grown in YPD for 5 days at 30 °C or 37 °C. The stability of R1 in KS-R1 was determined by the ratio of colonies on SC-Ura plates to those on YPD plates. The stability of R1 in KS-R1E was measured by the percentage of colonies maintaining all seven position markers via PCR. Values represent the mean ± standard deviation (SD) (*n* = 3).

As described in our prior work [27], chromosome 1 was circularized to obtain R1 by removing the telomeres and joining the ends with *KmURA3* using CRISPR/Cas9. During the circularization, the telomere-associated long repetitive sequence on chromosome 1 was also deleted to prevent homologous recombination [29]. To ensure effective selection of R1, the R1 carries three selective markers: *hyg^R^*, *kan^R^*, and *URA3* (Figure 1A). When KS-R1 grows in non-selective YPD medium, the loss rate of R1 is low, at only 0.11% per generation [27]. However, to maintain R1 during long-term culturing or under stress conditions, it is necessary to add two antibiotics (hygromycin and G418) to a nutrient-deficient medium (SC-Ura) for screening, which increases cultivation costs. Moreover, R1 occupies three common genetic markers, potentially affecting further engineering of KS-R1.

To stabilize R1 without relying on selective markers, we plan to utilize the essential genes on R1 to functionally compensate for the deletion of their counterparts in the *K. marxianus* genome. Currently, the essential genes of *K. marxianus* have not been fully annotated; therefore, we inferred these essential genes based on those present on R1. In total, there are 12 *S. cerevisiae* essential genes on R1 [30]. We compared the protein sequence identity and expression levels between these essential genes and their *K. marxianus* orthologs (Figure 1B). From this comparison, we selected three *K. marxianus* genes for deletion: *KmPOP5*, *KmPRP45*, and *KmMAK16*. ScMak16 and KmMak16 share a high identity and a low evolutionary rate, suggesting that their functions are likely conserved. *KmPOP5* and *KmPRP45* exhibited expression levels similar to those of *ScPOP5* and *ScPRP45*, indicating that the regulatory mechanisms of *POP5* and *PRP45* are relatively conserved. In addition, since *KmPOP5* and *KmPRP45* are adjacent in the *K. marxianus* genome, both genes can be deleted simultaneously, which simplifies the engineering process.

First, we sequentially deleted the *hyg^R^* and *kan^R^* markers on R1 using CRISPR. The deletion of these two markers was confirmed by PCR (Figure 1C). The resultant strain was named KS-R1-Δhk. Subsequently, we simultaneously deleted *KmPOP5* and *KmPRP45* in the KS-R1-Δhk strain (Figure 1C). The resultant strain was named KS-R1-Δhkpp. Next, we deleted *KmMAK16* in the KS-R1-Δhkpp strain, resulting in KS-R1-Δhkppm (Figure 1C). During the construction of R1, we installed direct repeats flanking the *URA3* gene [27]. Recombination between these direct repeats would remove the *URA3* marker. Therefore, we used 5-FOA to counterselect *ura3* mutants in KS-R1-Δhkppm. The loss of *URA3* in the positive clone was validated by PCR (Figure 1C). Subsequently, we confirmed that the engineered R1 retained all seven position markers (Figure 1C). These results indicate that removing all three selective markers from R1 did not affect its integrity. The resultant strain was named KS-R1E (E for “maintenance by essential genes”).

When a Km essential gene is deleted, its function is provided by its ortholog on R1. Therefore, the growth status of the engineered strains reflects the extent of functional complementation between the Km gene and its Sc ortholog. To investigate this, we compared the growth curves of the KS-R1 strain with those of KS-R1-Δhkpp, KS-R1-Δhkppm, and KS-R1E in YPD medium. The results showed that, compared to KS-R1, the initial growth rates of KS-R1-Δhkpp, KS-R1-Δhkppm, and KS-R1E were slightly slower. However, in the later phases, the biomass of all three engineered strains was close to that of KS-R1 (Figure 1D). These findings indicate that *ScPOP5*, *ScPRP45*, and *ScMAK16* can effectively compensate for the deletion of their Km counterparts, thereby maintaining the normal growth of the hybrid yeast.

In KS-R1E, all selective markers on R1 have been removed. The maintenance of R1 relies on functional compensation for the deletion of essential Km genes by their counterparts on R1. Theoretically, this compensation can maintain R1 without additional selective pressure. To verify this idea, we measured the chromosome stability of R1 in KS-R1 and KS-R1E in non-selective YPD medium (Figure 1E). The results showed that at 30 °C, the stability of R1 in KS-R1 was 91.9% after 5 days (approximately 35 generations), consistent with our previous reports [27], while the loss rate of R1 was zero in KS-R1E. At 37 °C, the stability of R1 in KS-R1 was 28.7% after 5 days, indicating that high temperature causes unstable inheritance of R1. In contrast, the loss rate of R1 in KS-R1E remained zero. These results indicate that the strategy of essential gene complementation can efficiently maintain R1 under both normal and stress conditions.

### 3.2. Characterization of Gene Loci on R1 Displaying Stable Transcriptional Activities

**Figure 2:**
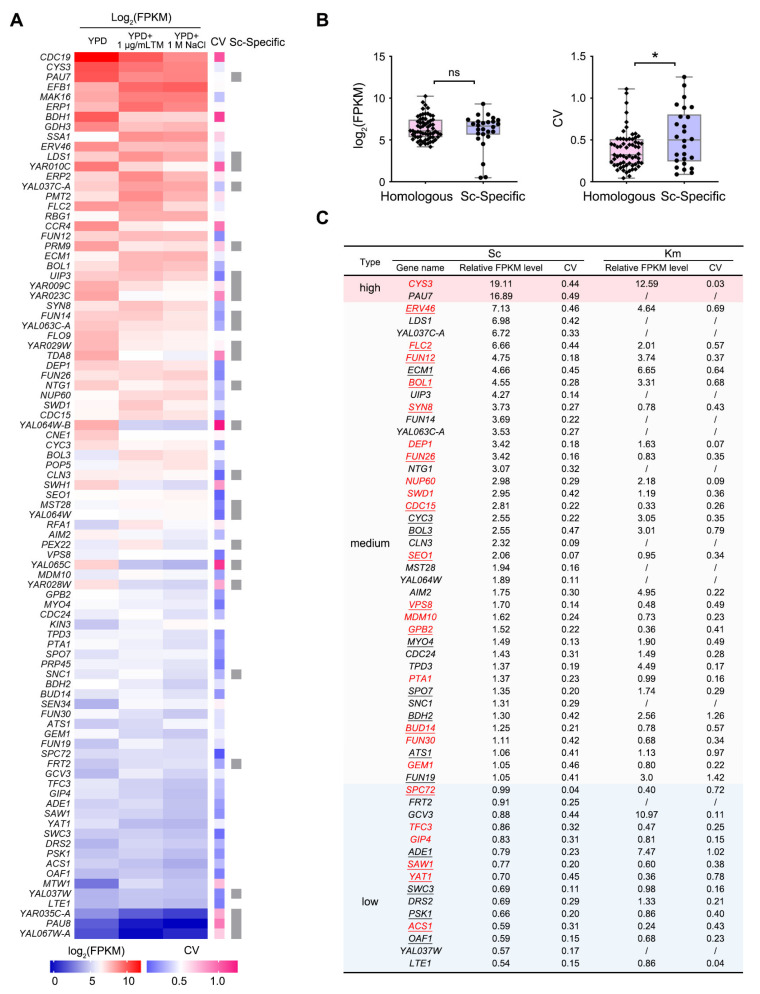
Characterization of gene loci on R1 displaying stable transcriptional activity. (**A**) Expression levels of R1 genes under various conditions. Log_2_-based FPKM values of R1 genes in YPD medium, YPD containing 1 μg/mL tunicamycin (TM), or YPD containing 1 M NaCl are displayed using color scales. The coefficients of variation (CV) of FPKM across these three conditions for each R1 gene are shown using a separate color scale. Sc-specific genes are indicated by gray blocks. (**B**) Comparison of the expression levels and their CVs for R1 genes with or without orthologous genes. ns, not significant; *: *p* < 0.05. Expression levels of an R1 gene are presented as the log_2_-based average FPKM value across three conditions. (**C**) List of R1 genes exhibiting stable expression across the three conditions. The “relative FPKM level” refers to the ratio of the average FPKM value of an R1 gene across three conditions to the average FPKM value of three housekeeping genes (*SWC4*, *TRK1*, *MPE1*) across the same conditions. R1 genes that displayed higher relative FPKM levels than their Km orthologs are labeled in red. R1 genes that displayed lower CV than their Km orthologs are underlined.

To identify suitable sites on R1 for gene insertion, we analyzed the transcriptomic data of R1 genes under three conditions: YPD medium, YPD containing 1 μg/mL tunicamycin, and YPD containing 1 M NaCl (Figure 2A). Among the 89 genes, 63 genes have orthologs in *K. marxianus*, while the remaining 26 genes are considered Sc-specific genes. Km and Sc orthologs were identified using OrthoVenn3 [32]. The expression levels of orthologous R1 genes were not significantly different from those of Sc-specific genes (Figure 2B). To identify the gene loci exhibiting stable transcriptional activities, the coefficients of variation (CV) of expression levels across the three conditions for each R1 gene were calculated (Figure 2A). The CVs of Sc-specific genes were significantly higher than those of orthologous R1 genes. This might be due to the faster divergence of *cis*-elements in Sc-specific genes, leading to greater fluctuations in gene expression under various conditions (Figure 2B).

We focused on genes that maintained stable expression across the three conditions (CV < 0.5), as these gene loci might offer consistent expression for inserted genes. Among 63 orthologous R1 genes, 46 genes (73%) were stably expressed. Among the Sc-specific genes, 13 genes (50%) were stably expressed, yielding a total of 59 stably expressed genes. Compared to the average expression level of three housekeeping genes (*SWC4*, *TRK1*, and *MPE1*), 2 of the stably expressed genes were high-expression genes (ratio > 10), 39 were medium-expression genes (1 < ratio < 10), and 15 were low-expression genes (ratio < 1) (Figure 2C). *ScPOP5*, *ScPRP45*, and *ScKmMAK16* are the remaining three stably expressed genes. Since they are required to maintain the growth of KS-R1E, their loci are not suitable for insertion and are not listed in Figure 2C. Therefore, the alien chromosome R1 contains 56 gene loci with varying and stable transcriptional activities, offering valuable insertion sites for multiple heterologous gene expressions.

Next, we compared the transcription levels and stability of the stably expressed R1 genes with their Km orthologs. Compared to the Km orthologs, 25 stably expressed R1 genes displayed higher expression levels (Figure 2C, labeled in red). This might be due to the inactivation of repressive elements of these R1 genes in the Km background, leading to subsequent derepression. Meanwhile, compared to their Km orthologs, 27 R1 genes exhibited greater stability, as reflected by their lower CV values (Figure 2C, underlined). In these R1 genes, stress-responsive *cis*-elements might not be functional in Km, resulting in stable expression under various stress conditions. These R1 gene loci are likely to provide more stable expression for inserted genes than the orthologous Km gene loci.

### 3.3. GFP Replacing R1 Genes at Different Loci Displayed Various Expression Levels

**Figure 3:**
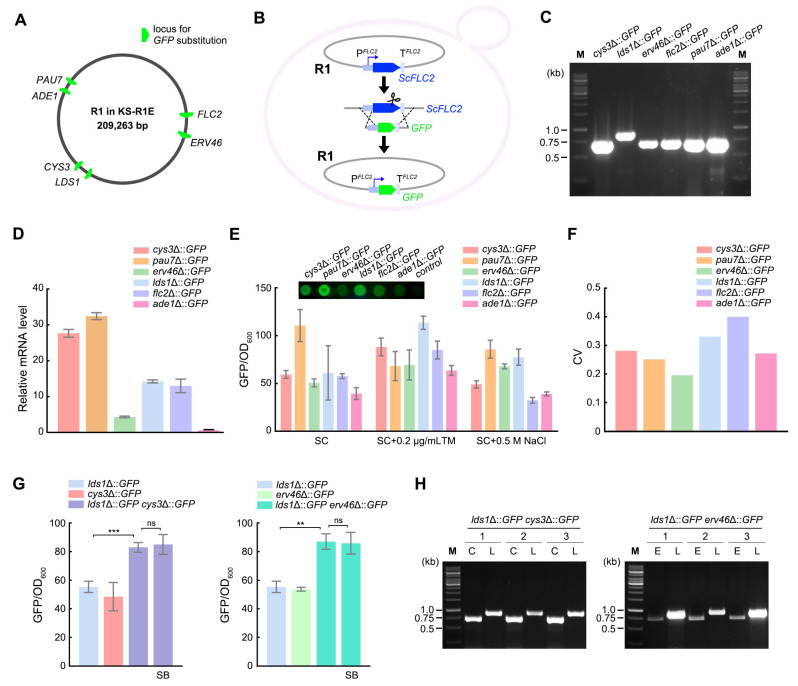
*GFP* replacing R1 genes at different loci displayed various expression levels. (**A**) Positions of five loci on R1 for *GFP* substitution. (**B**) Schematic view of replacing an R1 gene with *GFP* in situ. (**C**) Identification of substitution of R1 genes by *GFP* via PCR. The positions of primers relative to the loci and the expected sizes of the PCR products are shown in Figure S1B. (**D**) Relative mRNA levels of the *GFP* in KS-R1E strains with *GFP* integrated at different loci. Cells were collected after growing in YPD medium for 12 h. mRNA levels of the *GFP* were normalized to that of *KmSWC4*. Values represent the mean ± standard deviation (SD) (*n* = 3). (**E**) Intensity of GFP fluorescence at different loci under different conditions. Cells were grown overnight in SC medium, SC containing 0.5 M NaCl, or SC containing 0.2 μg/mL tunicamycin (TM). The fluorescence of the culture was measured and normalized by OD_600_. Values represent the mean ± SD (*n* = 3). A total of 5 OD cells grown in SC medium were spotted onto an SC plate, and the fluorescent image is shown above. (**F**) Coefficient of variation of GFP fluorescence at different loci across three conditions. (**G**) Intensity and stability of fluorescence in strains with *GFP* at two loci. Strains with *GFP* at two loci, as well as strains with *GFP* at a single locus, were grown overnight in SC medium, and fluorescence was measured. To assess stability, strains with *GFP* at two loci were subcultured in SC medium for 3 days, and the fluorescence of the subculture (SB) was measured. Values represent the mean ± SD (*n* = 3). **: *p* < 0.01, ***: *p* < 0.001, ns: not significant. (**H**) Identification of *GFP* integration after subculturing. One representative clone restreaked from the three parallel subcultures (1–3) in (**G**) was subjected to a PCR assay to validate the integration of *GFP* at the *LDS1* (L), *ERV46* (E), or *CYS3* (C) loci. The positions of primers relative to the loci and the expected sizes of the PCR products are shown in Figure S1B.

To verify whether loci on R1 can serve as insertion sites for heterologous genes, we inserted *GFP* into six loci with varying transcriptional activities: *CYS3* and *PAU7* (strong), *ERV46*, *FLC2*, and *LDS1* (medium), and *ADE1* (weak) (Figure 3A). We constructed a *GFP* donor containing the *GFP* ORF flanked by 500 bp of upstream and downstream sequences of the target R1 gene ORF. KS-R1E was transformed with a plasmid co-expressing gRNA and Cas9 along with the *GFP* donor. Through recombination between the donor and the R1 loci, the *GFP* ORF replaced the target gene ORF in situ. Subsequently, *GFP* expression was driven by the native promoter and terminator of the targeted R1 gene (Figure 3B).

We verified *GFP* insertion using one primer upstream of the targeted locus and another primer within the *GFP* ORF. Positive transformants yielded PCR bands of the expected size (Figure 3C). Next, we measured the mRNA levels of *GFP* expressed from different loci. In YPD medium, the mRNA levels of *GFP* expressed from the *CYS3*, *PAU7* (strong), *ERV46*, *FLC2*, and *LDS1* (medium) loci were higher than those at the *ADE1* (weak) locus (Figure 3D). The mRNA level of *GFP* expressed from the *CYS3* locus was lower than that from *PAU7*, and the mRNA level of *GFP* expressed from *ERV46* was lower than that from *LDS1* and *FLC2* (Figure 3D). This pattern differs from the ranking of transcriptional levels at these loci shown in Figure 2C. This discrepancy might be due to the fact that the transcriptional level of a locus is influenced not only by the promoter and terminator but also by mRNA sequence characteristics, such as mRNA stability [33,34]. Subsequently, we measured the intensity and stability of GFP fluorescence under different conditions (Figure 3E). SC medium was used in these experiments to reduce the GFP background signal observed in YPD medium. In SC medium, the ranking of GFP fluorescence intensities from different loci was consistent with the ranking of their mRNA levels (Figure 3E). The differences in GFP intensity between strains could be observed directly using a fluorescence microscope (Figure 3E, upper panel). Notably, the coefficient of variation (CV) of GFP levels across the six loci ranged from 0.2 to 0.4 under the three conditions, reflecting stability in expression (Figure 3F). These results demonstrate that R1 loci can achieve stable expression of inserted genes at varying levels.

In *K. marxianus*, transcriptional activities of gene loci with strong promoters, such as *TEF3*, *PDC1*, or *HXT4*, are 44~488 times the activity of the housekeeping *SWC4* locus in YPD [21,27]. In comparison, *CYS3*, *PAU7*, *ERV46*, *LDS1,* and *FLC2* loci exhibited transcriptional activities 34, 31, 13, 5, and 11 times that of the *SWC4* locus in YPD, respectively. Therefore, a single locus on R1 might not be sufficient to achieve the required level of overexpression. To address this issue, a heterologous gene can be inserted at multiple stable loci to achieve higher expression. To validate this idea, in the strain with *GFP* integrated at the *LDS1* locus, an additional *GFP* gene was integrated at either the *CYS3* or *ERV46* locus. The fluorescence intensity of strains with *GFP* at two loci was significantly higher than that of strains with *GFP* at a single *LDS1* locus, reflecting a roughly additive effect (Figure 3G). Next, the stability of *GFP* loci in strains carrying dual *GFP* was measured, since recombination might occur between identical ORFs. Strains were subcultured in SC medium for 3 days (approximately 21 generations), and the fluorescence intensity of the culture remained the same as that of the original culture, suggesting that both *GFP* copies were conserved (Figure 3G). We also examined the status of *GFP* loci in three clones restreaked from each parallel subculture. The results showed that *GFP* integrated at the *LDS1*, *CYS3*, and *ERV46* loci was retained in all clones after subculturing. The results for one representative clone are shown in Figure 3H. These findings indicate that the integration of the same ORF at two loci improved expression levels without readily causing rearrangement of R1.

### 3.4. Substitution of R1 Genes by P. pastoris Disulfide Bond Formation Genes Improved the Expression of Heterologous Proteins

**Figure 4:**
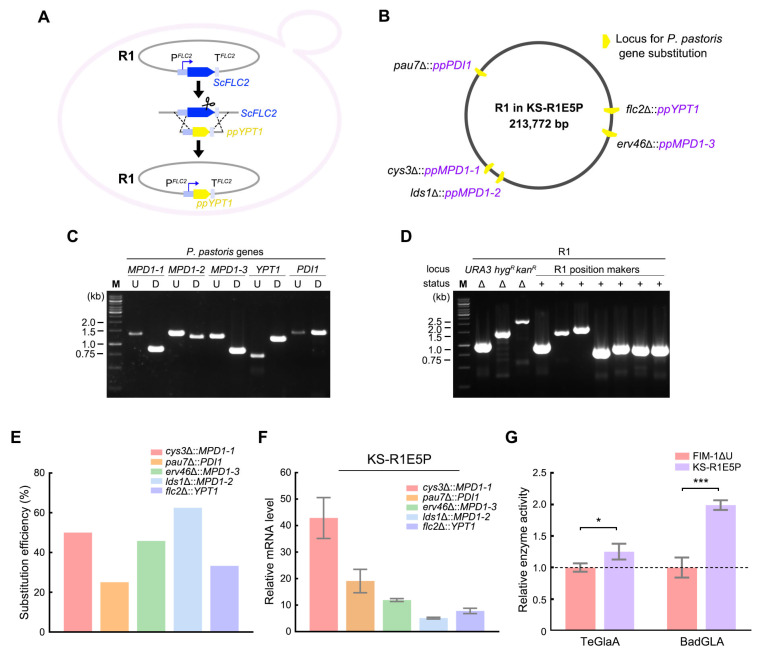
Substitution of R1 genes by *P. pastoris* disulfide bond formation genes improved the expression of heterologous proteins. (**A**) Schematic view of replacing an R1 gene in situ with *P. pastoris* disulfide bond formation genes. (**B**) Map of R1 after substitution of five R1 genes with *P. pastoris* genes. (**C**) Identification of the substitution of R1 genes by *P. pastoris* genes via PCR. The positions of primers relative to the loci and the expected sizes of the PCR products are shown in Figure S1C. U, upstream primer pair; D, downstream primer pair. (**D**) Validation of position markers and deletion of selective markers on R1 in KS-R1E5P. (**E**) Efficiency of substitution at the different loci. Efficiency was calculated as the percentage of positive clones among 24 total clones. (**F**) Relative mRNA levels of the *P. pastoris* genes in KS-R1E5P. Cells were collected after growing in YPD medium for 12 h. mRNA levels of the *P. pastoris* genes were normalized to that of *KmSWC4*. Values represent the mean ± SD (*n* = 3). (**G**) Comparison of the activities of BadGLA and TeGlaA expressed by KS-R1E5P and FIM-1ΔU. Cells were grown in YD medium for 72 h, and activities in the supernatant were measured. The average activity in FIM-1ΔU was designated as 1. Values represent the mean ± SD (*n* = 3). *: *p* < 0.05, ***: *p* < 0.001.

To verify whether loci on R1 can be used for the co-expression of multiple genes, we sequentially replaced five R1 genes with five *P. pastoris* disulfide bond formation genes using a strategy similar to the *GFP* substitution (Figure 4A). The ORFs of *CYS3*, *LDS1*, *ERV46*, *PAU7*, and *FLC2* were replaced with the ORFs of *PpMPD1-1*, *PpMPD1-2*, *PpMPD1-3*, *PpPDI1*, and *PpYPT1*, respectively (Figure 4B). Successful replacement of the R1 genes by *P. pastoris* genes was verified by PCR (Figure 4C). This engineering did not impair the integrity of R1, as R1 retained all the position markers and deletion of selective markers (Figure 4D). The substitution efficiencies at the *CYS3*, *PAU7*, *FLC2*, *ERV46*, and *LDS1* loci were 50%, 25%, 33.3%, 45.8%, and 62.5%, respectively (Figure 4E), indicating that these loci can stably integrate different heterologous genes. The resulting strain was named KS-R1E5P.

We performed qPCR to measure the expression levels of the *P. pastoris* genes in KS-R1E5P. The results showed that the expression levels of *PpMPD1-1*, *PpMPD1-2*, *PpMPD1-3*, *PpPDI1*, and *PpYPT1* were, respectively, 43, 5, 12, 19, and 8 times those of the control *KmSWC4* gene (Figure 4F). Notably, the relative mRNA level of the *P. pastoris* gene expressed from the *CYS3* or *ERV46* locus was higher than that of *GFP* mRNA expressed from the same locus (Figure 3D), reflecting the influence of the ORF sequence on mRNA levels.

Finally, we tested the effects of the insertion of multiple genes on the expression of heterologous proteins. We compared the expression of two glucoamylases, BadGLA and TeGlaA, in the FIM-1ΔU and KS-R1E5P strains. BadGLA and TeGlaA were predicted to contain one and four pairs of disulfide bonds, respectively [8]. These structural features require a robust disulfide bond formation machinery, making them ideal candidates for evaluating the impact of co-expressing *P. pastoris* genes involved in disulfide bond formation. Genes encoding BadGLA or TeGlaA were carried by a multicopy plasmid containing a *URA3* marker. Because KS-R1E5P did not occupy the *URA3* marker, it readily accepted these plasmids. The results showed that the secretory activities of BadGLA and TeGlaA in KS-R1E5P were significantly higher than those in FIM-1ΔU (Figure 4G). These findings indicate that the alien chromosome from *S. cerevisiae* can serve as a novel platform to provide sites for the expression of multiple heterologous genes, thereby enhancing the performance of the host.

## 4. Discussion

Our results indicate that an alien chromosome can serve as a novel platform for the expression of multiple heterologous genes. Compared to inserting heterologous genes into the genome, integrating them onto an alien chromosome does not disrupt the native genomic structure and avoids affecting the expression of nearby native genes. Plasmids and artificial chromosomes are two other options for heterologous gene insertion, but alien chromosomes offer greater stability. In this study, we maintained the alien chromosome using functional compensation between essential genes on the alien chromosome and deletion of their orthologs in the genome, resulting in a near-zero loss rate of the alien chromosome in non-selective conditions. The KS-R1E strain containing the modified alien chromosome, compared to the parental KS-R1 strain, showed a slightly reduced initial growth rate. However, biomass between KS-R1E and KS-R1 in the later growth phase was comparable. This result suggests that although the common ancestor of *S. cerevisiae* and *K. marxianus* diverged 115 million years ago [35], the regulatory expression and protein functions of Sc genes such as *POP5*, *PRP45*, and *MAK16* remain highly conserved with their Km counterparts, allowing these Sc orthologs to effectively compensate the deletion of their counterparts. In future research, replacing *ScPOP5*, *ScPRP45*, and *ScMAK16* on R1 with the corresponding Km genes may achieve complete functional compensation, and the slight growth defect of KS-R1E is expected to be redeemed.

Regardless of whether multiple heterologous genes are expressed via genome integration, plasmids, or artificial chromosomes, each gene requires suitable promoters and terminators. A major advantage of an alien chromosome is that it provides a series of loci with varying transcriptional activities, along with their native *cis*-regulatory elements. Typically, a gene cassette for replacement through recombination is composed of an upstream homologous arm, a promoter, a heterologous gene ORF, a terminator, and a downstream homologous arm [36]. On an alien chromosome, the regions flanking gene ORFs are natural promoters and terminators, so the composition of the gene cassette simplifies to: upstream homologous arm, heterologous gene ORF, downstream homologous arm (Figure 3B and Figure 4A). Our results showed that after in situ replacement of R1 genes with *P. pastoris* genes, the original transcriptional activities of the loci were almost fully recapitulated (Figure 4F). These findings demonstrate the effectiveness of R1 loci in providing differential expression of multiple genes.

This study focused on loci with stable expression levels. In addition to these stable loci, some genes show altered expression under specific stress conditions—for example, *KIN3* is specifically induced by NaCl, while *RFA1* and *PEX22* are specifically highly expressed in the presence of tunicamycin (Figure 2A). These loci might be used for condition-dependent expression. 

In summary, alien chromosomes not only provide suitable insertion sites for multi-gene expression in the *K. marxianus* but also offer a variety of *cis*-expression control elements like promoters and terminators with different activities. This new platform facilitates the engineering and optimization of *K. marxianus* in metabolic engineering and synthetic biology.

## Data Availability

The original contributions presented in the study are included in the article/Supplementary Material, further inquiries can be directed to the corresponding authors.

## Supplementary Materials

The following supporting information can be downloaded at: https://www.mdpi.com/article/10.3390/microorganisms13030509/s1, Table S1: Strains used in this study; Table S2: Plasmids used in this study; Table S3: Primers used in this study; Figure S1: The Positions of primers.
