# Supplementary material for: Alien Chromosome Serves as a Novel Platform for Multiple Gene Expression in Kluyveromyces marxianus
Source: Microorganisms. 2025 Feb 25;13(3):509. doi: 10.3390/microorganisms13030509 (PMC11946330; doi:10.3390/microorganisms13030509)
Supplement: Supplementary file 1 [file microorganisms-13-00509-s001.zip › Supplementary_Figure S1.pdf]

**A**

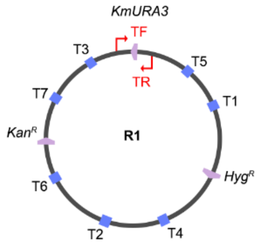


| Locus | Length_Intact (bp) | Length_deleted (bp) |
|---|---|---|
| *URA3* | 3323 | 1103 |
| *Hyg*$^R$ | 3184 | 1528 |
| *Kan*$^R$ | 3652 | 2228 |
| T1 | 990 | / |
| T2 | 1391 | / |
| T3 | 1583 | / |
| T4 | 836 | / |
| T5 | 924 | / |
| T6 | 888 | / |
| T7 | 900 | / |

**B**

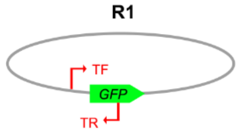


| Locus | Length (bp) |
|---|---|
| *cys3Δ::GFP* | 750 |
| *lds1Δ::GFP* | 912 |
| *erv46Δ::GFP* | 727 |
| *flc2Δ::GFP* | 729 |
| *pau7Δ::GFP* | 747 |
| *ade1Δ::GFP* | 755 |

**C**

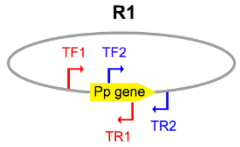


| Locus | Length_Up (bp) (TF1-TR1) | Length_Down (bp) (TF2-TR2) |
|---|---|---|
| *cys3Δ::MPD1-1* | 1349 | 868 |
| *lds1Δ::MPD1-2* | 1386 | 1224 |
| *erv46Δ::MPD1-3* | 1248 | 838 |
| *flc2Δ::YPT1* | 724 | 1167 |
| *pau7Δ::PDI1* | 1387 | 1410 |

**Figure S1.** The Positions of primers. (**A**) Primers used in constructing KS-R1E. The positions of these primers relative to the loci and the expected sizes of the PCR products are shown. (**B**) Primers used for replacing R1 genes with *GFP*. (**C**) Primers used for replacing R1 genes with *P. pastoris* genes.
